# Increased Hunger, Food Cravings, Food Reward, and Portion Size Selection after Sleep Curtailment in Women Without Obesity

**DOI:** 10.3390/nu11030663

**Published:** 2019-03-19

**Authors:** Chia-Lun Yang, Jerry Schnepp, Robin M. Tucker

**Affiliations:** 1 Department of Food Science and Human Nutrition, Michigan State University, East Lansing, MI 48824, USA; yangch39@msu.edu; 2 College of Technology Architecture and Applied Engineering, Bowling Green State University, Bowling Green, OH 43402, USA; schnepp@bgsu.edu

**Keywords:** sleep, hunger, food cravings, food reward, portion size

## Abstract

This study examined the effects of one night of sleep curtailment on hunger, food cravings, food reward, and portion size selection. Women who reported habitually sleeping 7–9 h per night, were aged 18–55, were not obese, and had no sleep disorders were recruited. Sleep conditions in this randomized crossover study consisted of a normal night (NN) and a curtailed night (CN) where time in bed was reduced by 33%. Hunger, tiredness, sleep quality, sleepiness, and food cravings were measured. A progressive ratio task using chocolates assessed the food reward. Participants selected portions of various foods that reflected how much they wanted to eat at that time. The sleep duration was measured using a single-channel electroencephalograph. Twenty-four participants completed the study. The total sleep time was shorter during the CN (*p* < 0.001). Participants reported increased hunger (*p* = 0.013), tiredness (*p* < 0.001), sleepiness (*p* < 0.001), and food cravings (*p* = 0.002) after the CN. More chocolate was consumed after the CN (*p* = 0.004). Larger portion sizes selected after the CN resulted in increased energy plated for lunch (*p* = 0.034). In conclusion, the present study observed increased hunger, food cravings, food reward, and portion sizes of food after a night of modest sleep curtailment. These maladaptive responses could lead to higher energy intake and, ultimately, weight gain.

## 1. Introduction

Insufficient sleep is an independent risk factor for overweight and obesity [1], and interventional studies demonstrate greater food intake after a night of curtailed sleep [2] or total sleep deprivation (TSD) [3]. Insufficient sleep, like obesity [4], is a global problem. Although the recommended sleep duration for adults is 7–9 h per night [5], approximately 1 in 3 American adults report sleeping <7 h per night [6], almost half of Japanese adults sleep <7 h [7], and nearly 1 in 3 Taiwanese adults sleep <6 h [8]. Given the large-scale public health implications, the mechanisms by which insufficient sleep increases obesity risk deserve further study.

There are a number of maladaptive changes that occur under conditions of insufficient sleep that could promote an excess intake leading to weight gain. For example, feelings of hunger increase after a night of TSD in males [9]. Increased ratings of self-reported sleepiness were associated with increased cravings for high-fat sweet foods [10]. Food cravings reflect a strong desire to consume foods that are palatable; these cravings typically involve sugary, high-fat foods [11]. An increased susceptibility to food reward under conditions of TSD has also been observed [12]. Food reward is a measure of the temporary value of food as it is being consumed [13], and an increased susceptibility to food reward has been associated with an increased intake [13].

In addition to the mechanisms promoting the excess intake described above, increases in self-selected portion size merit consideration. The amount of energy consumed at an eating occasion is largely determined by both the type of food and the amount of food selected (portion size) [14]. Importantly, when people serve themselves, over 90% of individuals consume the entire portion they self-select [14]. Portion size is influenced by expected satiety, which represents how filling the food is expected to be [15]. Expected satiety is dependent on the memory of previous experiences with specific foods [16]. Because insufficient sleep has been associated with poor performance on a variety of memory-related tasks [17], an exploration of the effects of insufficient sleep on self-selected portion size is warranted.

A considerable amount of work that has examined the physiological, psychological, and behavioral changes under conditions of insufficient sleep has relied on TSD, which does not reflect the real-world experiences of most individuals [18]. Another popular approach is to subject all participants to a prescribed amount of time in bed, for example, 4 h. Due to natural awakenings during the night, this means that the actual total sleep time will be less than the prescribed time in bed. The time in bed approach results in an uneven curtailment; longer sleepers receive a more severe curtailment than shorter sleepers, which could obscure findings if the study population was skewed towards shorter sleepers or inflate effects if the study population was comprised predominantly of longer sleepers. One way to address the ecological validity issue of TSD and the uneven application of curtailment is to reduce the habitual sleep time by a percentage. Based on the work of others [18,19], a 33% reduction was selected, which was projected to translate into a 2–3 h reduction.

The purpose of the current study was to examine the effects of a more modest sleep curtailment, taken as a percentage reduction of habitual sleep time, on factors shown to promote increased intake. These factors included: hunger sensations, food cravings, susceptibility to food reward, and self-selected portion size of foods representing a variety of sweet, savory, healthy, and unhealthy attributes. We hypothesized that all dependent variables would increase under conditions of sleep curtailment.

## 2. Materials and Methods

### 2.1. Participants

Participants included women without obesity (BMI < 30 kg/m^2^) between the ages of 18–55 who reported typically sleeping 7–9 h per night. The height and body weight were measured. The body fat was assessed using a bioelectrical impedance analysis (TBF-400, TANITA Corporation of America Inc., Arlington Heights, IL, USA). Potential participants were screened for possible sleep problems using the Pittsburgh Sleep Quality Index (PSQI) [20]. Participants had to score ≤5, suggesting no sleep problems [20]. Also, participants had to indicate that they enjoyed chocolate candy by rating their liking as 5 or more on a 10-point scale. Participants who indicated they did not have a usual bedtime were excluded. This was done to assist with the individualized sleep curtailment assignments. Given the fact that individuals with obesity can differ in terms of sleep architecture [21], sleep less [22] experience greater frequency of food craving [23], and differ in their responsivity to a food reward [24], participants were limited to individuals without obesity. The study was approved by the Human Research Protection Program (HRPP) at Michigan State University (East Lansing, MI, USA). All participants signed a consent form before testing.

### 2.2. Study Design

This randomized crossover study consisted of two different sleep conditions lasting one night each. Visits occurred at least two weeks apart. During the normal night (NN), participants went to bed and woke at their usual time. The habitual sleep time was based on the self-reported habitual duration collected using the PSQI questionnaire. Sleep was curtailed by 33% during the curtailed night (CN). This reduction was selected based on previous work noting such a reduction was more ecologically valid than more extreme curtailment [18,19]. The curtailment was split evenly between going to bed later and waking earlier to center the sleep and minimize circadian rhythm disruption due to curtailment [2,25]. Individualized instructions were given to participants to achieve the 33% curtailment. For example, if the participant’s usual sleep duration was from 10 p.m.–7 a.m., the bed time was changed to 11:30 p.m. and wake time was changed to 5:30 a.m. Confirmation of compliance to the curtailment protocol was done using the Zmachine (see Section 2.3).

Each visit occurred at the participant’s usual lunchtime and at the same time for each visit. Participants were instructed to consume the same breakfast and snack(s) at the same time on each testing day. Researchers verified the food that participants had in the morning by asking them to recall their intake and to confirm their compliance. The researchers did not restrict these foods. If the participants did not follow the diet instructions, they were asked to reschedule the visit. Participants were instructed not to eat or drink for at least one hour before the visit. Upon arrival at the lab, participants completed questionnaires (see Section 2.4, Section 2.5, Section 2.6 and Section 2.7) and consumed 200 mL of water to minimize sensations of thirst contributing to hunger sensations.

### 2.3. Sleep Measurement

The night before each visit, participants wore a single-channel electroencephalography (EEG) device (Zmachine, General Sleep, Cleveland, OH). The Zmachine has been shown to substantially agree with polysomnography (PSG), the gold standard of sleep measurement [26]. The Zmachine allows participants to sleep at home and is less invasive than PSG. For this study, the Zmachine recorded time in bed (TIB), total sleep time (TST), slow wave sleep (SWS), and rapid eye movement (REM) sleep and was used for sleep measurements in both the NN and CN conditions. The percentages of SWS and REM were calculated by dividing each stage’s duration by TST.

### 2.4. Questionnaires—Hunger, Tiredness, Sleep Quality, and Sleepiness Measures

Visual analog scales (VAS) assessed hunger and tiredness (scored as 0 = not at all, 100 = extremely), as well as the quality of sleep for the previous night (0 = much worse than normal, 100 = much better than normal). Immediately prior to testing, current feelings of sleepiness were assessed using the validated Karolinska Sleepiness Scale (KSS) [27]. The KSS asks about the degree of sleepiness during the previous 5 min. Scores range from 1 (extremely alert) to 9 (very sleepy).

### 2.5. Food Cravings

The validated General Food Cravings Questionnaire-State (G-FCQ-S) was used to measure current cravings for general, rather than specific, foods [11]. The questionnaire has five subscales: an intense desire to eat, anticipation of relief from negative states and feelings as a result of eating, anticipation of positive reinforcement that may result from eating, obsessive preoccupation with food or lack of control over time, and craving as a physiological state. Responses range from 1 to 5. The higher the score, the stronger the sensation of craving.

### 2.6. Portion Size Testing and Liking of Food

Chicken breast, white rice, salad, salad dressing, and soda were provided as meal options. Potato chips, mini chocolate sandwich cookies, green seedless grapes, and gummy candies were provided as snacks in order to provide a variety of food options. Participants were presented with large containers of food and were told “Based on your appetite right now, please prepare a meal using these meal options. Please select only the foods you wish to eat. You’re not actually going to eat what you select. This is just an exercise.” Amounts plated rather than consumption were measured in order to eliminate the effects of palatability and post-ingestive experiences on portion size selection during the subsequent visit [28,29]. Participants indicated if they drank diet or regular soda. In addition, participants were told, “Please choose which of these snacks you would like to eat right now. You can choose more than one. Please put your snacks into separate bowls.” Participants were provided with two 26.5 cm (10.5 in) white plates, one 730 mL (24.7 oz) glass, one 118.3 mL (4 oz) plastic cup, and four 15.3 cm (6 in) white bowls to use. Researchers weighed all selected food items to a nearest 0.1 g. The manufacturer’s information was used to analyze the energy and macronutrient content of the selections. Individual food liking was tested at the first visit to evaluate whether the test foods were liked by the participants or not. Participants were asked to indicate “How much do you like to eat (the food)?” on a VAS (0 = not at all, 100 = very much).

### 2.7. Food Reward

Food reward can be quantified by asking participants their desire to eat, as well as measuring the amount of money they are willing to pay or work they perform to access a food [13]. Therefore, food reward was assessed using two different methods. First, participants were asked to indicate the amount of money they were willing to pay for a standardized serving (pay-for-food) [13]. Based on the MyPlate website [30] and the manufacturer’s information, one serving of each food was presented: 71 g white rice (0.5 cup), 83 g salad (2 cups), 28.5 g chicken breast (1 oz), 30 g salad dressing (2 tablespoons), 29 g mini chocolate sandwich cookies (9 pieces), 39 g gummy candies (17 pieces), 28 g potato chips (1 oz), and 147 g grapes (1 cup). Participants indicated their payment amount on a scale between 0–10 U.S. dollars and with the instruction of “Imagine you are having this food for lunch today. What is the maximum you would pay for this much food? [31]” The second method used to assess food reward utilized a progressive ratio task [13] where participants pressed a computer mouse button in exchange for chocolate candies (work-for-chocolate). The instructions said “You can earn a reward by clicking on the button on the next screen. Click as much or as little as you like. When you no longer want to continue, press the ‘Quit’ button to stop the session.” Participants were instructed to eat each candy that was earned before continuing and could stop after eating the first candy or continue working for more. Food reward tasks were completed after the portion size testing.

### 2.8. Statistical Analysis

A data analysis was conducted using IBM SPSS 25.0 statistical software (IBM Corporation, Armonk, NY, USA). Data are reported using means and standard deviations. Portion size information is presented using calories (kcals). Paired *t*-tests with bootstrapping [32] were used to test for differences between the normal and curtailed nights. A *p*-value under 0.05 was considered significant. Sleep staging is reported for 22 of the 24 participants due to Zmachine malfunction. Self-reported TIB was used for these participants.

## 3. Results

### 3.1. Participants

One-hundred and thirteen women were screened to identify 44 eligible participants. Of these participants, 14 were excluded due to an inability to follow the sleep or dietary protocol, three participants withdrew due to difficulties sleeping with the Zmachine, and three participants withdrew due to scheduling issues. Twenty-four participants completed the study. Participant characteristics are shown in Table 1.

### 3.2. Sleep Time, Hunger, Tiredness, Sleep Quality and Sleepiness

The sleep curtailment protocol was effective at significantly reducing the amount of sleep the participants obtained on the CN compared to the NN (4.60 ± 0.72 vs. 7.03 ± 0.96 h; *p* < 0.001) (Table 2). TIB and TST were reduced by 33.5% and 34.6% during the CN, respectively (*p* < 0.001, for both). SWS and REM sleep were lower during the CN compared to the NN (*p* < 0.001, for both). The percentage of SWS was higher in CN compared to NN (*p* = 0.012). Compared to the NN, participants reported increased sensations of hunger, tiredness, and sleepiness, and poorer sleep quality after the CN (*p* < 0.05, for all) (Table 3).

### 3.3. Food Cravings, Food Reward for Test Food

The total G-FCQ-S scores were significantly higher after the CN (Table 4). All subscales except “anticipation of positive reinforcement that may result from eating” were also significantly higher. For the work-for-chocolate task, significantly more chocolate candies were consumed after the CN (CN: 3.3 ± 1.5 vs. NN: 2.6 ± 0.9, *p* = 0.004). There was no difference between NN and CN on the pay-for-food task.

### 3.4. Selected Portion Size of Food and Macronutrient Content

**Figure 1:**
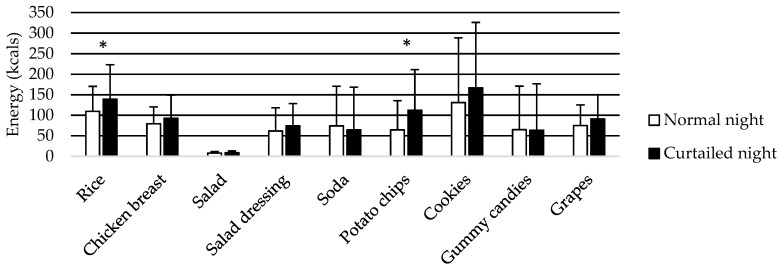
Means and standard deviations of selected portion sizes of meals and snacks after the normal night and curtailed night. The amount of energy selected from rice and potato chips was significantly higher after the curtailed night. * *p* < 0.05.

Participants liked all foods (all scores > 50 (neutral)) except for the gummy candies and soda, which had a rating of 44.9 and 32.5, respectively. Apart from these two foods, all other foods were liked equally, with the exception that grapes were liked more than potato chips (*p* = 0.034). Portion size was significantly larger after the CN than the NN for white rice (139.3 ± 83.9 vs. 109.7 ± 60.7 kcal, *p* = 0.014) and potato chips (112.4 ± 99.0 vs. 64.5 ± 71.1 kcal, *p* = 0.030) (Figure 1). The portions of all other foods, with the exception of gummy candies and soda, were larger after the CN, although not significantly.

After the CN, participants chose more protein, fat, and total calories from the meal-associated foods compared to the NN (Table 5). Selections from snack-associated foods were higher in fat after the CN (Table 5). In terms of macronutrients from the total food selected (combining meals and snacks), protein, fat, and the percentage of total calories from fat were significantly higher after the CN than after the NN (Table 5).

## 4. Discussion

Insufficient sleep is associated with an increased energy intake [2] and an increased risk of obesity [1]. This study evaluated the effect of modest sleep curtailment on hunger, food cravings, food reward, and portion size, all of which have been shown to contribute to excess intake and possible weight gain, but previous interventional studies have used more extreme curtailments [2] or even total deprivation [3,12,33]. The modest curtailment in this study resulted in significantly reduced TIB, TST, SWS, and REM sleep durations as well as reduced subjective sleep quality. The percentage of REM and SWS ranged from ~21–~29% during both nights, which is consistent with healthy sleep [34]. The reduction in sleep duration resulted in participants feeling hungrier, reporting both increased food cravings and increased susceptibility to food reward, and selecting larger portions from meal items during lunch time, even though the same amount of breakfast foods and snacks were consumed at the same time on both days.

Despite the fact that participants consumed the same amount of food at the same time each day, hunger at lunchtime was increased, as was the portion size of selected foods and the energy content of meal-associated foods after sleep curtailment. These findings are consistent with other research that reported that TSD led to higher hunger ratings after TSD compared to after a normal night’s sleep in men [3,9]. These studies also observed that concentrations of the hunger hormone ghrelin increased, which could contribute to feelings of increased hunger. Others have also reported increased ghrelin as well as decreased leptin after a sleep restriction of four hours for four days [35]. Not only is such a maladaptive change in hormones capable of influencing appetitive sensations, it can also affect food consumption as higher ghrelin and lower leptin levels are frequently associated with a higher energy intake [35]. Unlike a previous study in nine men where hunger did not differ between a night of longer (7 h) compared to a night of shorter sleep (4.5 h) [9], we observed higher hunger ratings in our all-female sample. Whether insufficient sleep affects appetitive sensations differently by sex warrants further investigation. Taken together, these findings suggest that insufficient sleep results in increased hunger and the selection of more energy, in part due to an appetitive hormone dysregulation; however, it should be noted that the question of changes in appetitive hormones under conditions of insufficient sleep is not settled; others have observed that after five days of five-hour sleep curtailment, ghrelin concentrations decreased while leptin concentrations increased [36]. Clearly, further work is needed to resolve these discrepancies.

The total food craving scores on the G-FCQ-S were higher after the CN compared to after the NN, suggesting that participants had an increased urge to eat palatable foods. Higher food cravings have been associated with excess energy intake and with obesity [11], although people do not always give in to cravings [37]. Previous work noted that sleepiness is related to food cravings for both savory and sugary high-fat foods [10], and individuals who reported a short sleep duration (<7 h) reported higher cravings for high-calorie foods [38]. Thus, evidence suggests that individuals who experience modest sleep curtailment may also experience increased food cravings, which could promote increased energy intake in susceptible individuals.

Food reward was higher after the sleep curtailment for the work-for-chocolate task, but no differences in the pay-for-food task were observed. It could be the case that a more severe curtailment is necessary to observe differences for both measures. Changes in the brain activity associated with the food reward have been noted after insufficient sleep [33]. Previous research demonstrated that TSD increased the willingness of men to pay more for food [12]. That study also observed changes in the hypothalamus activity associated with food reward. A separate study noted that sleep deprivation increased activity in the right anterior cingulate cortex, which is associated with an increased appetite for energy-dense food [33]. These findings suggest that insufficient sleep increases brain activity when individuals are exposed to food, which likely explains our findings of an increased willingness to engage in the work-for-chocolate task.

Larger portions are associated with increased food consumption [39]. Previous work reported that people establish appropriate meal sizes based on experience [14]. This experience plays an important role in determining portion size, more so than hunger [40]. Still, self-selected portion sizes are subject to variation [3,35,41]. Insufficient sleep is known to impair the ability to pay attention [17], and eating while distracted can lead to increased portion sizes and an increased intake [42]. This could be a mechanism by which sleep affects portion size selection. In terms of lunch options, participants plated 12.4% more total calories after the CN. Given the high correlation between self-selected portion sizes and intake [14], this result suggests that even modest sleep curtailment may result in an increased energy intake. The dietary intake for the rest of the day was not measured, so it is not known if dietary compensation occurs after a larger lunch. However, previous work demonstrated that while energy expenditure increases after insufficient sleep, energy intake overwhelms that increase, resulting in a net positive energy balance [36].

Macronutrient content was significantly different for total food selected (combining meals and snacks) after the CN. Calories from fat and total percent calories from fat were significantly higher after the CN, which agrees with previous work demonstrating that sleep curtailment led to higher fat consumption in adolescents [43]. In addition to fat content, the present study also observed that participants plated more protein from meal foods and from all foods after the CN than after the NN. Protein is considered to be the most satiating macronutrient and can postpone the feeling of hunger longer when compared to carbohydrate and fat [44]. Whether changing the macronutrient content at meals counteracts the effects of a curtailed night of sleep warrants future testing.

This study did not observe significant differences between the two sleep conditions for energy selected from snacks. This finding is inconsistent with previous work that noted that after TSD or sleep curtailment, participants selected larger portions of snacks [3,35,45]. Since the present study asked participants to choose snack foods at lunchtime, if participants did not consider snack foods appropriate for lunch, differences might be less likely. However, the present study observed that participants took more chips after sleep curtailment, which led to increased fat selected among the snack options. Potato chips are a high-fat, savory food, and previous work suggests that the intake of these foods is higher among adolescents who slept less than 8 h per night compared to those who slept more than 8 h per night [46]. The fact that potato chips are often consumed during meals as well as during snacks could explain the increased selection. Regardless, these results suggest that sleep curtailment led individuals to choose a high-fat, palatable food that resulted in increased energy coming from fat.

The strengths of this study included the randomized design to minimize order effects, use of objective sleep measurements rather than self-reporting, matching the time and content of breakfast to avoid confounding, centering the sleep curtailment to minimize circadian rhythm disruption, and conducting study visits at the same time two weeks apart to reduce the risk that participants would remember their responses between visits. There are several limitations to the present study. First, the actual food intake was not measured. However, self-selected portions resulted in more than 90% of participants cleaning their plates in a previous study [14]. Habitual sleep patterns were obtained from self-reports, which can be unreliable, although others have reported moderate correlations between subjective and objective measures of sleep duration (*r* = 0.45) [47]. Only one night’s worth of sleep data for each sleep condition was obtained, which obscures the effects of a possible accumulation of sleep debt; however, we purposefully recruited “good” sleepers with habitual bedtimes in order to minimize night-to-night variability. While menstrual cycles were not measured, randomization should have minimized the effects of menstruation on the study outcomes. Our female-only sample limits generalizability as the effects of insufficient sleep may differ by sex [48]. While breakfast was not standardized, diet recalls were used to confirm that each day’s breakfast was the same. It is possible that the additional time awake led to increased energy expenditure leading to increased hunger; however, the timing of meals did not differ between days, so this effect is likely minimal but warrants further exploration.

## 5. Conclusions

While previous studies utilized severe sleep curtailments or even complete deprivation, the present study observed increased hunger, food cravings, food reward, and larger selected portion sizes under a more modest 33% sleep reduction. Taken together, these responses and behaviors could contribute to an increased intake and, ultimately, weight gain. Future work should focus on how these maladaptive responses to sleep curtailment can be addressed to prevent excess intake and weight gain.

## Figures and Tables

**Table 1:** Characteristics of participants (*n* = 24).

Variable	Mean (SD)
Age (year)	24.4 (7.2)
BMI (kg/m^2^)	22.1 (2.6)
Body fat (%)	25.8 (6.7)
PSQI	3.1 (1.1)
Race	*n* (%)
White	18 (75.0)
Asian	6 (25.0)
Ethnicity	*n* (%)
Non-Hispanic	23 (95.8)
Prefer not to answer	1 (4.2)

SD, standard deviation; BMI, body mass index; PSQI, Pittsburgh Sleep Quality Index.

**Table 2:** Sleep parameters from the Zmachine.

Sleep Parameter	Normal Night	Curtailed Night	*p*-Value
TIB (hours)	8.19 (0.66)	5.45 (0.56)	<0.001
TST (hours)	7.03 (0.96)	4.60 (0.72)	<0.001
SWS (hours)	1.49 (0.41)	1.15 (0.41)	<0.001
REM sleep (hours)	2.03 (0.74)	1.30 (0.48)	<0.001
SWS (%)	21.19 (5.05)	24.67 (7.84)	0.012
REM sleep (%)	28.57 (8.91)	28.00 (9.10)	0.800

Data expressed as: mean (SD); TIB: time in bed; TST: total sleep time; SWS: slow wave sleep; REM: rapid eye movement.

**Table 3:** Effects of curtailed sleep on self-reported sleepiness, tiredness, quality of sleep and hunger.

	Normal Night	Curtailed Night	*p*-Value
Sleepiness ^1^	2.8 (1.3)	4.9 (1.9)	<0.001
Tiredness ^2^	24.8 (16.2)	58.5 (15.3)	<0.001
Quality of sleep ^2^	55.2 (17.2)	43.0 (17.0)	0.030
Hunger ^2^	53.7 (16.9)	60.8 (15.7)	0.013

Data expressed as: Mean (SD); ^1^ Assessed by Karolinska Sleepiness Scale (score of 1–9); ^2^ Assessed by visual analog scales (mm).

**Table 4:** Effects of curtailed sleep on food cravings and food reward.

	Normal Night	Curtailed Night	*p*-Value
**Food cravings**			
Total G-FCQ-S	45.5 (8.4)	51.5 (7.4)	0.002 *
Factor I	9.5 (2.3)	11.0 (1.9)	0.009 *
Factor II	9.7 (2.3)	11.1 (2.1)	0.008 *
Factor III	9.9 (1.5)	11.2 (1.8)	0.009 *
Factor IV	6.3 (2.4)	7.7 (2.5)	0.022 *
Factor V	10.0 (2.2)	10.6 (1.9)	0.236
**Food reward**			
Chocolate count (each)	2.6 (0.9)	3.3 (1.5)	0.004 *

Data expressed as: Mean (SD); G-FCQ-S, General Food Cravings Questionnaire-State. Factor I: An intense desire to eat; Factor II: Anticipation of relief from negative states and feelings as a result of eating; Factor III: Craving as a physiological state. Factor IV: Obsessive preoccupation with food or lack of control over eating; Factor V: Anticipation of positive reinforcement that may result from eating. * *p* < 0.05.

**Table 5:** Energy and percentage of total energy by macronutrient for normal night sleep and curtailed night sleep.

	Meal			Snack			Total		
Energy (kcal)	NN	CN	*p*-Value	NN	CN	*p*-Value	NN	CN	*p*-Value
Carbohydrate	179.4 (108.4)	194.7 (124.4)	0.295	239 (144.7)	294.2 (131.8)	0.106	418.4 (207.8)	488.9 (195)	0.068
Sugar	84.8 (95.4)	75.4 (102.6)	0.406	73.9 (61.6)	84.4 (70)	0.515	158.7 (128.3)	159.8 (143.4)	0.960
Protein	72.2 (32)	85.3 (44.2)	0.046 *	10.4 (8.4)	13.7 (9.4)	0.144	82.7 (32.8)	99 (47.1)	0.035 *
Fat	81.1 (53)	99.8 (55.4)	0.008 *	85.8 (74.6)	126.3 (85.7)	0.036 *	166.9 (114.6)	226.1 (115.2)	0.006 *
Total kcal	332.7 (139.5)	379.8 (170)	0.034 *	335.2 (212.5)	434.2 (206.8)	0.064	667.9 (302.7)	814.0 (321.3)	0.200
Carbohydrate (%)	50.5 (17.5)	48.2 (16.6)	0.339	71.8 (12.9)	70.4 (11)	0.516	61.9 (10.5)	60.5 (8)	0.312
Protein (%)	24.5 (12.7)	24.4 (14.1)	0.970	2.9 (1.2)	2.9 (1)	0.976	13.8 (5.4)	12.6 (5.7)	0.247
Fat (%)	24.9 (11.7)	27.3 (11)	0.096	25.3(12.6)	26.7 (10.7)	0.500	24.2 (8.6)	26.9 (7.2)	0.014 *

Values expressed as Mean (SD); NN: normal night; CN: curtailed night. * *p* < 0.05.
